# Treatment of COVID-19-associated ARDS with umbilical cord-derived mesenchymal stromal cells in the STROMA-CoV-2 multicenter randomized double-blind trial: long-term safety, respiratory function, and quality of life

**DOI:** 10.1186/s13287-024-03729-w

**Published:** 2024-04-19

**Authors:** Alexandre Sitbon, Caroline Hauw-Berlemont, Miryam Mebarki, Nicholas Heming, Julien Mayaux, Jean-Luc Diehl, Alexandre Demoule, Djillali Annane, Clémence Marois, Sophie Demeret, Emmanuel Weiss, Guillaume Voiriot, Muriel Fartoukh, Jean‐Michel Constantin, Bruno Mégarbane, Gaëtan Plantefève, Hélène Boucher-Pillet, Guillaume Churlaud, Audrey Cras, Camille Maheux, Chloé Pezzana, Mamadou Hassimiou Diallo, Said Lebbah, Jacques Ropers, Joe-Elie Salem, Christian Straus, Philippe Menasché, Jérôme Larghero, Antoine Monsel, APHP STROMA–CoV‐2 Collaborative Research Group, Déborah Benchetrit, Harold Bonvallot, Fanny Charbonnier-Beaupel, Meriem Dhib-Charfi, Pierre Romain Delmotte, Assitan Kone, Marine Le Corre, Carole Metz, Louis Puybasset, Corinne Vezinet

**Affiliations:** 1 Multidisciplinary Intensive Care Unit, Department of Anesthesiology–Critical Care and Perioperative Medicine, La Pitié–Salpêtrière Hospital, Assistance Publique-Hôpitaux de Paris (APHP) Sorbonne University, 47–83, boulevard de l’Hôpital, 75651 Paris Cedex 13, France; 2 Intensive Care Unit, APHP-CUP, Hôpital Européen Georges-Pompidou, Université Paris Cité, 75015 Paris, France; 3 APHP, Hôpital Saint-Louis, Unité de Thérapie Cellulaire, Centre d’Investigation Clinique en Biothérapies CBT501, INSERM, Université Paris Cité, Paris, France; 4 FHU SEPSIS, Department of Intensive Care, Hôpital Raymond-Poincaré (APHP), Laboratory of Infection & Inflammation–INSERM U1173, Simone Veil School of Medicine, University Versailles Saint Quentin–University Paris Saclay, 92380 Garches, France; 5 APHP, Groupe Hospitalier Universitaire–Sorbonne Université, site Pitié–Salpêtrière, Service de Médecine Intensive et Réanimation (Département R3S), INSERM, UMRS1158 Neurophysiologie Respiratoire Expérimentale et Clinique, Sorbonne Université, Paris, France; 6 Innovative Therapies in Hemostasis, INSERM, Université Paris Cité, 75006 Paris, France; 7 Biosurgical Research Laboratory (Carpentier Foundation), APHP-CUP, Hôpital Européen Georges-Pompidou, 75015 Paris, France; 8 Neurological Intensive Care Unit, Department of Neurology, La Pitié–Salpêtrière Hospital, APHP, Sorbonne University, Paris, France; 9 Groupe de Recherche Clinique en REanimation et Soins Intensifs du Patient en Insuffisance Respiratoire aiguE (GRC-RESPIRE), Sorbonne Université, Paris, France; 10 Department of Anesthesiology and Critical Care, Beaujon Hospital, DMU PARABOL, APHP Nord, Paris, France; 11 Center for Research on Inflammation, INSERM, Université Paris Cité, Paris, France; 12 Centre de Recherche Saint-Antoine UMRS_938 INSERM, Assistance Publique – Hôpitaux de Paris, Service de Médecine Intensive Réanimation, Hôpital Tenon, Sorbonne Université, Paris, France; 13 Department of Medical and Toxicological Critical Care, Lariboisière Hospital, INSERM UMRS1144, University of Paris, Paris, France; 14 Service de Réanimation Polyvalente, Centre Hospitalier Victor Dupouy, 69, Rue du Lieutenant-Colonel Prud’hon, 95100 Argenteuil, France; 15 Centre MEARY de Thérapie Cellulaire et Génique, APHP, Hôpital Saint-Louis, Paris, France; 16 INSERM UMR1140, Université Paris Cité, 75006 Paris, France; 17 INSERM, UMR S 970, Paris Centre de Recherche Cardiovasculaire (PARCC), Université de Paris, Paris, France; 18 Clinical Research Unit, Pitié–Salpêtrière University Hospital, APHP, Paris, France; 19 Institut National de la Santé et de la Recherche Médicale (INSERM), Assistance Publique - Hôpitaux de Paris (AP-HP), Clinical Investigation Center (CIC-1901), Regional Pharmacovigilance Centre, Department of Pharmacology, Pitié-Salpêtrière Hospital, Sorbonne Université, Paris, France; 20 INSERM, UMRS1158 Neurophysiologie Respiratoire Expérimentale et Clinique; AP-HP, Groupe Hospitalier Universitaire APHP-Sorbonne Université, site Pitié-Salpêtrière, Département R3S (Respiration, Réanimation, Réadaptation Respiratoire, Sommeil), Service d’Explorations Fonctionnelles de la Respiration, de l’Exercice et de la Dyspnée, Sorbonne Université, 75013 Paris, France; 21 Department of Cardiovascular Surgery, Hôpital Européen Georges-Pompidou, Paris, France; 22 INSERM UMRS_959, Immunology–Immunopathology–Immunotherapy (I3), Sorbonne Université, 75013 Paris, France; 23 Biotherapy (CIC-BTi), Hôpital Pitié-Salpêtrière, APHP, 75651 Paris, France

**Keywords:** Severe acute respiratory syndrome coronavirus‐2, Acute respiratory distress syndrome, Umbilical cord‐ derived mesenchymal stromal cells, Long-term outcomes, Follow-up Studies, Quality of Life at six and twelve months after hospital discharge

## Abstract

**Background:**

The STROMA-CoV-2 study was a French phase 2b, multicenter, double-blind, randomized, placebo-controlled clinical trial that did not identify a significant efficacy of umbilical cord-derived mesenchymal stromal cells in patients with SARS-CoV-2-induced acute respiratory distress syndrome. Safety on day 28 was found to be good. The aim of our extended study was to assess the 6- and 12-month safety of UC-MSCs administration in the STROMA-CoV-2 cohort.

**Methods:**

A detailed multi-domain assessment was conducted at 6 and 12 months following hospital discharge focusing on adverse events, lung computed tomography-scan, pulmonary and muscular functional status, and quality of life in the STROMA-CoV-2 cohort including SARS–CoV-2-related early (< 96 h) mild‐to-severe acute respiratory distress syndrome.

**Results:**

Between April 2020 and October 2020, 47 patients were enrolled, of whom 19 completed a 1-year follow-up. There were no significant differences in any endpoints or adverse effects between the UC-MSCs and placebo groups at the 6- and 12-month assessments. Ground-glass opacities persisted at 1 year in 5 patients (26.3%). Furthermore, diffusing capacity for carbon monoxide remained altered over 1 year, although no patient required oxygen or non-invasive ventilatory support. Quality of life revealed declines in mental, emotional and physical health throughout the follow-up period, and the six-minute walking distance remained slightly impaired at the 1-year patient assessment.

**Conclusions:**

This study suggests a favorable safety profile for the use of intravenous UC-MSCs in the context of the first French wave of SARS-CoV-2-related moderate-to-severe acute respiratory distress syndrome, with no adverse effects observed at 1 year.

## To the editor

STROMA-CoV-2 was a phase 2b, multicenter, double-blind, randomized, placebo-controlled trial that showed no efficacy of Wharton’s jelly human umbilical cord-derived mesenchymal stromal cells (UC-MSCs) on the PaO_2_/FiO_2_-ratio change between day 0 and day 7 in 47 patients with SARS–CoV-2-induced acute respiratory distress syndrome (ARDS) compared to placebo [1], even though this ratio remained unchanged over this time frame in the placebo group while it increased in the cell-treated one. Repeated UC-MSCs infusions were not associated with any serious adverse events during treatment or thereafter (until day 28). Our long-term study aims to evaluate the safety of UC-MSCs by monitoring patients at 6 and 12 months post-treatment, focusing on adverse events, lung computed tomography (CT)-scan, functional assessment of pulmonary and respiratory muscular capacities, and quality of life. To evaluate these long-term results, we conducted a comprehensive multi-domain assessment 1 year post-hospital discharge.

## Methods

Adult patients with SARS-CoV-2-associated ARDS of less than 96 h duration (the onset of ARDS was defined as the day on which a positive diagnosis of ARDS was made according to the Berlin criteria), enrolled in the French multicenter STROMA-CoV-2 trial (3 × 10^6^ UC-MSCs/kg given in three intravenous injections at 48-h intervals versus placebo) [1], were followed up at 6 and 12 months post-hospital discharge. The trial was approved by the National Review Board of Île-de-France III (CNRIPH 20.03.26.39722) and authorized by the French National Agency for Medicines and Health Products Safety (EudraCT 2020-001287-28), with registration at ClinicalTrials.gov (identifier NCT04333368; Registered 29 March 2020; https://clinicaltrials.gov/study/NCT04333368?term=NCT04333368&rank=1).

At 6 and 12 months, a comprehensive multi-domain assessment was conducted, encompassing adverse events screening, pulmonary function tests (PFT) with lung volumes, spirometry, diffusing capacity, and respiratory muscle strength assessment (maximal inspiratory pressure (MIP), maximal expiratory pressure (MEP), sniff nasal inspiratory pressure (SNIP)), alongside patient-reported outcome measures of quality of life (36-Item Short Form Survey (SF-36) and EuroQol 5 Dimension 5 Level survey (EQ-5D)). CT-scans at both end-inspiration and end-expiration were evaluated by two chest radiologists for the presence of pulmonary abnormalities [2]. Physical performance was also assessed using the 6-min walking test (6-MWT) [3], during which dyspnea and perceived exertion were quantified using the Borg scale [4, 5].

Continuous variables were described using their means and standard deviations and with frequencies and percentages for categorical variables. Between groups comparisons were performed using Wilcoxon rank-sum test for continuous variables and with Fisher exact test for categorical variables. Quality of life scales variations from M6 to M12 were explored using MMRM (Mixed Models for Repeated Measures). 95% confidence intervals were calculated and *p* values below the 0.05 threshold were considered as significant. All calculations were performed using the R software.

## Main findings

**Fig. 1:**
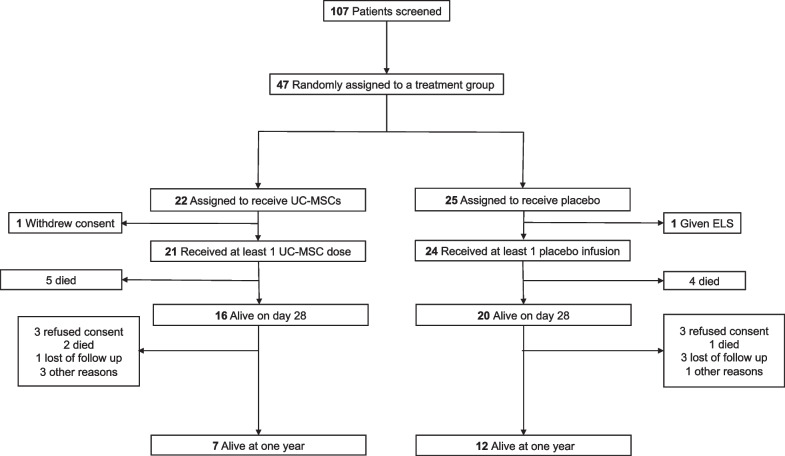
Flow chart of the trial. *ELS* extracorporeal life support. *UC-MSCs* umbilical cord-derived mesenchymal stromal cells

Between April and October 2020, 107 patients were admitted to the Intensive Care Units across the 10 participating centers and were assessed for study eligibility. Of these, 47 patients were successfully enrolled in the STROMA-CoV-2 cohort and 45 received at least one dose of UC-MSCs. Baseline characteristics upon admission were initially presented in the primary study [1]. Furthermore, the flow chart is reported in the Fig. 1.

**Fig. 2:**
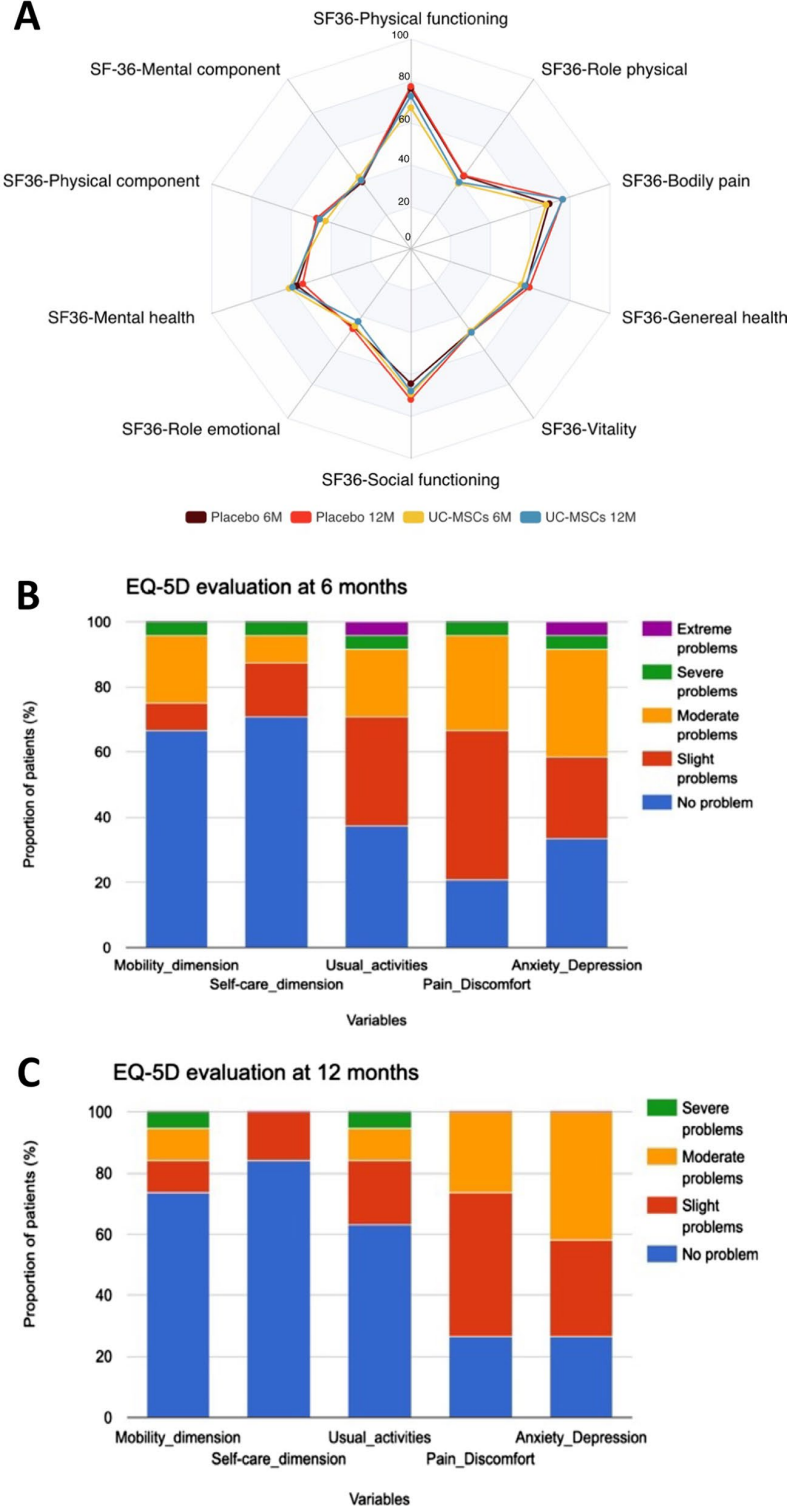
Comparative analysis of patient-reported outcomes using the medical outcome study 36-item short form survey and the EuroQol 5 dimension 5 level dimensions at 6 and 12 months. **A** UC-MSCs *versus* placebo groups are visualized as radar chart. Each of the eight domains is scored on a scale from 0 to 100, with a higher score indicating better health and less impact of health on usual roles. In this radar chart, the central point represents a score of 0, while the outermost boundary corresponds to a score of 100. **B** and **C** Bar chart representation depicting the EQ-5D variables at 6 months (**B**) and at 12 months (**C**). At the 6-month assessment, 11 patients from the UC-MSCs group and 13 from the placebo group were evaluated (24 patients in global cohort). At the 12-month mark, 7 patients from the UC-MSCs group and 12 from the placebo group were assessed (19 patients in global cohort). *EQ-5D* EuroQol 5 Dimension 5 Level*. M* month*. UC-MSCs* umbilical cord-derived mesenchymal stromal cells. *SF-36* 36-Item Short Form Survey

Findings are detailed in Tables 1, 2 and Fig. 2. Since the first part of the study was published covering follow-up to day 28 [1], we report in this second part of the study, 4 additional serious adverse events occurring in 3 patients (2 in the UC-MSCs group and 1 in the placebo group) from day 29 to 1 year of follow-up (Tables 1, 2). After 12 months of follow-up, the incidence of adverse events was similar between the groups, with 19 patients (90.5%) in the UC-MSCs group and 20 patients (83.3%) in the placebo group (*p* = 0.67). Likewise, the occurrence of serious adverse events was comparable, affecting 9 patients (42.8%) in the UC-MSCs group and 9 patients (37.5%) in the placebo group (*p* = 0.77).

**Table 1:** Comparative analysis of multi-domain outcomes at 6- and 12-month post-hospital discharge: UC-MSCs versus placebo

	6 months				12 months			
	UC-MSCs (n = 21)	Placebo (n = 24)	All cohort (n = 45)	P**	UC-MSCs (n = 21)	Placebo (n = 24)	All cohort (n = 45)	P**
Number of patients with adverse events [n (%)]	18 (85.7)	20 (83.3)	38 (84.4)	1.0	19 (90.5)	20 (83.3)	39 (86.7)	0.67
Number of patients with serious adverse events [n (%)]	9 (42.9)	9 (37.5)	18 (40.0)	0.77	9 (42.9)	9 (37.5)	18 (40)	0.77
Mortality [n (%)]	7 (38.9) Loss-to-follow-up N = 3	5 (27.8) Loss-to-follow-up N = 6	12 (33.3) Loss-to-follow-up N = 9	0.72	7 (50) Loss-to-follow-up N = 7	5 (29.4) Loss-to-follow-up N = 7	12 (38.7) Loss-to-follow-up N = 14	0.29

HRCT	N = 11	N = 13	N = 24		N = 7	N = 12	N = 19	
Lung nodules present [n (%)]	4 (36.4)	1 (7.7)	5 (20.8)	0.14	4 (57.1)	4 (33.3)	8 (42.1)	0.38
Groundglass present [n (%)]	7 (63.6)	4 (30.8)	11 (45.8)	0.22	3 (42.9)	2 (16.7)	5 (26.3)	0.30
Reticular opacities present [n (%)]	0 (0)	0 (0)	0 (0)	NA	0 (0)	1 (8.3)	1 (5.3)	1.0
Lung fibrosis present [n (%)]	1 (9.1)	2 (15.4)	3 (12.5)	1.0	0(0)	0 (0)	0 (0)	NA
Pleural effusion present [n (%)]	0 (0)	1 (7.7)	1 (4.2)	1.0	0(0)	0 (0)	0(0)	NA
Bronchi(ol)ectasis present [n (%)]	3 (27.3)	0 (0)	3 (12.5)	0.08	0 (0)	2 (16.7)	2 (10.5)	0.51

Pulmonary function test	N = 11	N = 13*	N = 24		N = 6	N = 6	N = 12	
TLC (L)	5.4 (1.3)	5.6 (1.3)	5.5 (1.3)	0.84	5.5 (1.5)	5.9 (0.7)	5.7 (1.1)	0.82
TLC (% of predicted)	85.8 (18.2)	91.9 (16.2)	89.1 (17.1)	0.35	89 (19)	85.7 (9.6)	87.3 (14.5)	0.94
RV (L)	2.6 (0.7)	2.86 (0.9)	2.7 (0.8)	0.62	2.5 (0.7)	2.9 (0.7)	2.7 (0.7)	0.59
RV (% of predicted)	78.1 (18.9)	89.6 (26.1)	84.3 (23.4)	0.26	78.8 (22.3)	79.5 (15.2)	79.2 (18.2)	0.81
FEV1 (L)	2.9 (0.8)	3.0 (0.6)	2.9 (0.6)	0.66	2.9 (0.9)	3.1 (0.7)	3.0 (0.8)	0.70
FEV1 (% of predicted)	96.6 (14.5)	100.9 (21.5)	98.9 (18.0)	0.43	99 (11.9)	96.7 (25.2)	97.8 (18.8)	0.70
FEV1/FVC (ratio)	83 (5)	78 (8)	80.0 (7)	0.07	80.7 (5.1)	77.8 (8.8)	79.2 (7)	0.52
FEV1/SVC (ratio)	81 (6)	77 (8)	79 (7)	0.34	80.3 (6.7)	73.5 (8)	76.9 (7.9)	0.11
SNIP (cmH_2_O)	82.2 (23.6)	70.2 (27)	75.6 (25.6)	0.30	84.3 (23.6)	84.2 (35.3)	84.3 (27.9)	1.00
SNIP (% of predicted)	87 (18.8)	73.1 (24.5)	79.3 (22.7)	0.21	92 (29.7)	84.2 (33.9)	88.5 (30.3)	0.78
MEP (cmH_2_O)	144.1 (56.8)	134.5 (53.9)	138.8 (54)	0.52	127.2 (43.9)	147.2 (66.4)	136.3 (53.2)	0.66
MEP (% of predicted)	76.9 (27.4)	77.1 (39.4)	77 (33.7)	0.50	70.7 (25.2)	89.6 (67)	79.3 (47)	1.00
MIP (cmH_2_O)	111.3 (36.9)	86.4 (29.9)	97.6 (34.7)	0.04	120.6 (30.3)	86.6 (30.3)	102.5 (17.1)	0.07
MIP (% of predicted)	114 (45.2)	91.5 (37.6)	101.7 (41.6)	0.24	131 (49.3)	76.6 (21.2)	103.8 (45.8)	0.10
DLCO (% of predicted)	71.2 (9.5)	70.8 (24.8)	71.0 (19.3)	0.66	67.5 (6.5)	67.3 (12.2)	67.4 (9.4)	0.69

Blood gas	N = 11	N = 11	N = 22		N = 5	N = 7	N = 12	
pH	7.44 (0.06)	7.4 (0.04)	7.42 (0.05)	0.05	7.43 (0.03)	7.41 (0.01)	7.42 (0.02)	0.19
PaO2 (mmHg)	88.9 (9.3)	93.6 (10.4)	91.2 (9.9)	0.14	94 (13.7)	89.29 (11.7)	91.3 (12.2)	0.63
PaCO_2_ (mmHg)	37.6 (6.4)	38 (3.46)	37.8 (5.0)	0.77	37 (3.7)	38.9 (4.5)	38.1 (4.1)	0.46
SaO_2_ (%)	97.27 (1.2)	97.64 (1.6)	97.5 (1.4)	0.17	97.6 (1.1)	97.1 (1.9)	97.3 (1.6)	0.86
HCO_3−_ (mmol/L)	25.36 (2.3)	23.45 (2.2)	24.4 (2.4)	0.10	24.8 (0.9)	24.3 (1.9)	24.5 (1.5)	0.67
Lactate (mmol/l)	1.37 (0.5)	1 (0.5)	1.2 (0.5)	0.13	1.3 (0.3)	1.1 (0.8)	1.2 (0.6)	0.17

Physical performance	N = 11	N = 11	N = 22		N = 6	N = 4	N = 10	
6MWT-Distance (m)	454.6 (101.6)	535.5 (67.9)	495 (94)	0.08	502.2 (81)	519 (22.7)	508.9 (62.4)	1.00
6MWT-Perceived exertion-BS	1.3 (2.1)	1.8 (2.8)	1.6 (2.4)	0.68	3.2 (2.2)	3.2 (3.9)	3.2 (2.9)	0.80
6MWT-Perceived dyspnea-BS	2.9 (2.8)	5 (3.3)	4 (3.2)	0.13	4.5 (1.9)	4.5 (3.9)	4.5 (2.6)	1.00
6MWT-SpO_2_ (%)	96.1 (3.3)	93.9 (4.2)	95 (3.8)	0.21	96 (4.5)	95.8 (5.4)	95.9 (4.6)	0.66
6MWT-Heart rate (bpm)	109.6 (13.9)	110.5 (24.5)	110.1 (19.7)	0.65	111.8 (7.6)	107.2 (14)	110 (10.1)	0.91

Data are presented as mean (standard deviation) or number (%), unless indicated otherwise. *BS* Borg scale. *bpm* beats per minute. *DLCO* diffusing capacity for carbon monoxide. *FEV1* forced expiratory volume in 1 s. *FVC* forced vital capacity. *HRCT* high-resolution computed tomography. *MEP* maximal expiratory pressure. *MIP* maximal inspiratory pressure. *RV* residual volume. *SNIP* sniff nasal inspiratory pressure. *Sp0*_*2*_ oxygen pulsed saturation. *SVC* slow vital capacity. *TLC* total lung capacity. *UC-MSCs* umbilical cord-derived mesenchymal stromal cells. *VAS* visual analogue scale. *VC* vital capacity. *6MWT* 6-min walking test

*In the placebo group, one patient did not undergo plethysmography

**Analyses were conducted comparing UC-MSCs and placebo at both six and twelve months using Wilcoxon rank-sum test for continuous data and Fisher exact test for categorical data

**Table 2:** Reported serious adverse events after day 28 up to 12 months of follow-up post-hospital discharge

	Group	Type of reaction/event	Relatedness of study to reaction/event	Outcome of reaction/event	Investigator’s comments
Patient 1 (event 1)	UC-MSCs	Coma	No	Death	No causality regarding this event
Patient 1 (event 2)	UC-MSCs	Terminal extubation	No	Death	No causality regarding this event
Patient 2	UC-MSCs	Septic shock	No	Death	Event is related to disease progression (ARDS associated to COVID-19)
Patient 3	Placebo	Multiple organ failure	No	Death	Event is related to disease progression (ARDS associated to COVID-19)

The nature of the adverse events (serious, see Table 2, and non-serious—data not shown) encountered in this second part of the study (follow-up from D29 to 12 months) was comparable to that of the adverse events reported in our first part of the study (follow-up to D28), and mostly related to the spontaneous evolution of severe COVID-19 disease in intensive care patients with a high severity score. Likewise, the distribution of the nature of these events was similar in the 2 groups (UC-MSCs versus *placebo*), with the majority of these adverse events relating to the evolution of severe COVID-19 disease (sepsis, septic shock, multiple organ failure). Significantly, no adverse events were reported that could be directly attributed to the investigational therapy, demonstrating the favorable safety profile of intravenous UC-MSCs in this context up to 1 year following the end of the treatment.

No differences were observed between the UC-MSCs and the placebo groups in terms of mortality, CT-scan lung morphology, lung and muscle function, gas exchange, 6-MWT performances or quality of life (Table 1 and Fig. 2) at 6 or 12 months after hospital discharge. Mixed models for repeated measures adjusted on age and sex also did not show any difference between UC-MSCs and *placebo* on either quality of life metrics (data not shown). At the study's end, there were 12 deaths, with 5 (29.4%) in the placebo group and 7 (50%) in the UC-MSCs group (*p* = 0.29). Although maximal inspiratory force (MIP) at 6 months was higher in the UC-MSCs than in the placebo group (mean ± standard deviation (SD)) 111.3 ± 36.9 versus 86.4 ± 29.9 cmH_2_O (*p* = 0.0439), this difference was no longer significant at 1 year with 120.6 ± 30.3 versus 86.6 ± 30.3 cmH_2_O, respectively (*p* = 0.0749). Importantly, no signs of fibrosis progression were detected at the 1-year assessment in any group. The variation in endpoints assessed from month 6 to month 12 also did not differ between the UC-MSCs and placebo groups, except for the variation in plasma bicarbonates levels, which had no clinical relevance (data not shown).

Featuring the evolution of patients with COVID-19-related ARDS, residual ground-glass opacities were observed in 11 patients (45.8%) and 5 patients (26.3%) at 6 and 12 months respectively, with no evidence of pro-fibrotic progression. Pulmonary function test results remained stable between 6- and 12-month assessments, with no significant alterations apart from a carbon monoxide diffusion capacity (DLCO) remaining slightly to moderately impaired at 6 and 12 months ((mean ± SD) 71.0 ± 19.3% and 67.4 ± 9.4% of expected theoretical values, respectively). Arterial blood gas analyses conducted at the 6-month and 1-year follow-up indicated the absence of chronic hypercapnia and no requirement of home oxygen therapy. Assessment of respiratory muscles strength showed that while MIP values remained within the normal range at 6 and 12 months, MEP and SNIP were recorded below the expected values (Table 1), suggesting a slight and persistent decline in respiratory muscle strength at 6 and 12 months following SARS-CoV-2-induced ARDS. The walking distance remained stable over time, with an average 6-MWT distance of (mean ± SD) 508.9 ± 62.4 m at 12 months, which remains slightly below the expected distance of (mean ± SD) 571 ± 90 m. [6]. The SF-36 domains with the most impaired scores at 6 months and 1 year were the physical component, the role physical, the mental component and the role emotional (Fig. 2). These data were confirmed by the EQ-5D score, characterized by a high incidence of mild-to-moderate anxiety and depressive symptoms present in 73.7% of patients at 1 year, matching the incidence of mild-to-moderate pain and discomfort (Fig. 2).

## Discussion

This study highlights a favorable safety profile associated with repeated intravenous administration of UC-MSCs up to 1 year after hospital discharge for COVID-19-associated ARDS, including adverse events, mortality, CT-scan imaging, pulmonary function tests including active muscle tests and quality of life scores. These safety results are consistent with previous clinical trials involving patients with COVID-19-associated ARDS, and showing a favorable safety profile of intravenous UC-MSCs [7, 8].

The incidence of diffuse lung ground-glass opacities persisting 1 year after hospitalization (26.3%) in the context of severe COVID-19-associated pneumonia is in line with the results of a meta-analysis including 3134 patients, in which 21.2% (95CI [15.4–28.4], I^2^ = 86.7%) presented with ground-glass opacities 1 year after hospitalization [9]. The absence of pulmonary fibrosis observed in our cohort contrasts, however, with the results of this meta-analysis reporting an incidence of fibrosis of 20.6% (95CI [1.0–35.2], I^2^ = 91.9%) [9]. While prolonged impairment of DLCO has been found in other studies, its persistence at 1 year is in contrast to some studies showing some degree of DLCO recovery between 6 months and 1 year [10]. While our study therefore confirms that the persistence of diffuse pulmonary opacities combined with prolonged impairment of DLCO appear to characterize the 1-year course of severe pneumonia associated with COVID-19, the small number of patients included, their inclusion during the first wave and the absence of corticosteroid therapy in our study could explain these discrepancies. Regarding the results of the 6-MWT test, our results mirror those found after non-COVID-related ARDS, with an estimated value of 422 m 1 year after ARDS [11], which remains below the values expected in healthy subjects [12]. This impairment therefore does not appear to be specific to COVID-19-associated pneumonia. Results for quality-of-life parameters mirror those observed in a cohort of patients with COVID-19 treated with corticosteroids [13]. Regarding the SF-36, mental, emotional and physical components were the most impaired, as previously reported [14]. Among the five categories of the EQ-5D, anxiety and pain were the most reported, corroborating the findings of previously published research [15], including studies of non-COVID ARDS [16]. Thus, the degree of impairment of quality of life of patients found at 1 year in our study does not seem to diverge from that already reported at 1 year following non-COVID-19 ARDS.

The main limitations of our study are the small sample size, the absence of baseline endpoints and an inclusion selecting severe patients from the first pandemic wave, with a high incidence of invasive mechanical ventilation. Similarly, the absence of patients vaccinated and/or treated with corticosteroids or other immunotherapies may have affected the findings.

## Conclusion

This study demonstrates a favorable safety profile of repeated intravenous 3 × 10^6^ UC-MSCs/kg in the context of the first French wave of COVID-19-associated moderate-to-severe early ARDS, with no adverse effects observed at 6 and 12 months after hospital discharge. The persistence at 1 year of lung opacities combined with impaired DLCO does not appear to lead to greater functional impairment than that observed 1 year after non-COVID-19 ARDS.

## Data Availability

Qualified clinical researchers can request access to de-identified participant dataset, informed consent forms and related documents, including the study protocol that underlie this article through submission of a proposal with a valuable research question to the corresponding author, subject to agreement of a contract.

## Abbreviations

ARDS: Acute respiratory distress syndrome
CI95: Confidence interval at 95%
COVID-19: Coronavirus disease-2019
CT: Computed tomography,
D: Day
DLCO: Diffusion capacity of the lung for carbon monoxide
MEP: Maximal expiratory pressure
MIP: Maximal inspiratory pressure
PaO_2_/FiO_2_: Partial pressure of oxygen to fractional inspired oxygen
PFT: Pulmonary function tests
ICU: Intensive care unit
IQR: Interquartile range
MSCs: Mesenchymal stromal cells
SARS-COV-2: Severe acute respiratory syndrome coronavirus-2
SD: Standard deviation
SNIP: Sniff nasal inspiratory pressure
SOFA: Sequential Organ-Failure Assessment
UC: Umbilical cord
UC-MSCs: Umbilical cord-derived mesenchymal stromal cells
